# Nanoantioxidant–Based Silica Particles as Flavonoid Carrier for Drug Delivery Applications

**DOI:** 10.3390/pharmaceutics12040302

**Published:** 2020-03-26

**Authors:** Francisco Arriagada, Germán Günther, Javier Morales

**Affiliations:** 1 Instituto de Farmacia, Facultad de Ciencias, Universidad Austral de Chile, 5110033 Valdivia, Chile; 2 Facultad de Ciencias Químicas y Farmacéuticas, Universidad de Chile, 8380494 Santiago, Chile; ggunther@ciq.uchile.cl

**Keywords:** drug delivery, flavonoid, silica nanoparticles, nanocarrier, nanoantioxidant, morin, rosmarinic acid

## Abstract

Nanosystems used in pharmaceutical formulations have shown promising results in enhancing the administration of drugs of difficult formulations. In particular, porous silica nanoparticles have demonstrated excellent properties for application in biological systems; however, there are still several challenges related to the development of more effective and biocompatible materials. An interesting approach to enhance these nanomaterials has been the development of nanoantioxidant carriers. In this work, a hybrid nanoantioxidant carrier based on porous silica nanoplatform with rosmarinic acid antioxidant immobilized on its surface were developed and characterized. Techniques such as dynamic light scattering (DLS), zeta potential, transmission electron microscopy (TEM), N_2_ adsorption–desorption measurements, differential scanning calorimetry (DSC), Fourier transform–infrared spectroscopy (FT-IR), and 2,2-diphenyl-1-picrylhydrazyl (DPPH^●^) assay were used to characterize and evaluate the antioxidant activity of nanocarriers. In addition, drug release profile was evaluated using two biorelevant media. The antioxidant activity of rosmarinic acid was maintained, suggesting the correct disposition of the moiety. Kinetic studies reveal that more morin is released in the simulated intestinal fluid than in the gastric one, while an anomalous non–Fickian release mechanism was observed. These results suggest a promising antioxidant nanocarrier suitable for future application in drug delivery.

## 1. Introduction

Great advancements in the area of nanomaterials have brought about opportunities to improve various processes in fields related to engineering, food industry, biomedicine, and the pharmaceutical industry, among others [1,2,3]. In particular, nanosystems used in pharmaceutical formulations, biomedical devices, or in theranostics have shown promising results in enhancing the administration of drugs of difficult formulations, decrease in therapy time, and early diagnosis of diseases [4]. In this context, porous silica nanoparticles have been studied as drug delivery systems (DDS) in the last two decades [5]. However, despite its excellent properties, such as its biocompatibility, high specific surface area and versatility on its surface functionalization, several challenges remain. Some of them are related to toxicity in certain tissues, clinical translation, industrial scaling, and optimization in specific drug delivery in the desired therapeutic target, in order to be incorporated as pharmaceutical products [6,7,8,9]. To overcome some of these drawbacks, bio-inspired, bio-mimetic, and smart materials, among others, have been developed [10,11,12]. An emerging strategy has been the development of systems called nanoantioxidants [13,14]. These systems are nanoparticles with intrinsic antioxidant activity and/or antioxidant-functionalized nanoparticles, in order to stabilize or improve the antioxidant activity, and produce materials with synergistic effects and more biocompatible, with applications as pharmaceutical excipients, food packaging or pharmaceutical carriers [15,16,17]. A relatively simple way to develop a polyphenolic layer on nanoparticles surface is by physical adsorption. Nonetheless, in this manner, the antioxidant molecule could undergo easy oxidation or polymerization, e.g., polyphenols browning, which restricts its lifetime; in addition, unwanted desorption and undesirable interaction could reduce its antioxidant function [16,17]. Therefore, the metal-phenolic networks approach [18] or covalent immobilization could overcome these drawbacks, generating a better pharmaceutical excipient (for non-porous particles) or, in the case of carrier particles, a better pharmaceutical vehicle. The latter ones, in general, are hybrid materials inspired by the carrier properties of inorganic nanoplatforms with functionalized antioxidants on the surface. Nevertheless, reports in the literature on its use as vehicles for drug delivery are limited. For example, Massaro and co-workers developed a synergistic nanoantioxidant based on the grafted of trolox on the surface of halloysite nanotubes loaded with quercetin [19]. In another study, Ebabe Elle and co-workers reported a novel carrier using mesoporous silica nanoparticles functionalized with caffeic acid or rutin, showing that the level of ROS generated by the bare silica nanoparticles decreases with the immobilization of these polyphenols on the surface of nanoparticles [20]. Arriagada and colleagues developed core-shell mesoporous silica nanospheres functionalized with caffeic acid, showing the ability of the antioxidant nanomaterial to protect oxidation-sensitive molecules [21]. These types of nanoantioxidant attempt to generate more effective and biocompatible materials, not only by using natural polyphenols to coat nanoparticles but also by releasing biomolecules with potential pharmacological activity, such as flavonoids. 

Morin (3,5,7,2′,4′-pentahydroxyflavone) (MH) is a flavonoid that commonly occurs in various fruits and vegetables of the *Moraceae* family [22,23], which has been shown a remarkable antioxidant [24,25], anti-inflammatory [26], neuroprotective [27], and anti-cancer activity [28] in different animal models. Thus, its oral administration could have promising health benefits; however, its applications are limited since morin is a BCS class IV molecule of difficult pharmaceutical formulations [29,30], which has very low oral bioavailability; studies suggest that in order to improve it, the intestinal first-pass effect should be avoided [31]. Nonetheless, selecting MH as a molecule probe to study its release behavior from a nanoantioxidant carrier and evaluating the impact that the surface polyphenolic layer of this nanosystem has on the release mechanism remains a challenge in pharmaceutical interest, which seeks to optimize potential formulations that include these novel nanoantioxidants. As far as we know, there are no reports on drug release from silica-based antioxidant systems. Therefore, the goal of this work was to synthesize surface-functionalized mesoporous silica nanoparticles with an antioxidant molecule to study the incorporation of morin and its subsequent release. To achieve the above, rosmarinic acid (RA), an ester of caffeic acid and 3,4-dihydroxyphenyllactic acid [32,33] with several promising biological activities, such as anti–inflammatory [34], anti–diabetic [35], prevention of Alzheimer’s disease [36], and potent antioxidant activity [32,33], was chosen to immobilize it onto mesoporous silica nanoparticles. Thus, RA could maintain its remarkable antioxidant activity due to both caffeoyl and 2-oxyphenylpropanoyl moieties, which allows the generation of a hybrid antioxidant nanocarrier. All the materials were properly characterized and the antioxidant activity of the system evaluated. To elucidate the release mechanism, different kinetic models were used.

## 2. Materials and Methods 

### 2.1. Materials

Morin hydrate (MH, ≥85%), rosmarinic acid (RA, ≥98%), tetraethyl orthosilicate (TEOS, 98%), (3-aminopropyl)triethoxysilane (APTES, ≥98%), hexadecyltrimethylammonium chloride solution (CTAC, 25 wt.%), *N*-(3-dimethylaminopropyl)-*N*′-ethylcarbodiimide hydrochloride (EDC, ≥98%), *N*-Hydroxysuccinimide (NHS, 98%), 3Å molecular sieves, 2,2-Diphenyl-1-picryl-hydrazyl (DPPH^●^), trifluoroacetic acid (TFA, ≥99%), and pancreatin from porcine pancreas (USP specifications) were purchased from Sigma-Aldrich (St. Louis, MO, USA). A dialysis bag (SnakeSkin^®^ Dialysis Tubing, molecular weight cutoff (MWCO) of 10,000 Da) was purchased from Thermo Scientific (Rockford, IL, USA). Ethanol (HPLC grade), sodium hydroxide (NaOH, ≥98%), acetic acid (AcOH, acs reagent for analysis), hydrochloric acid fuming (HCl, acs reagent 37%), acetonitrile (HPLC grade), methanol (HPLC grade), and pepsin from porcine gastric mucosa (0.7 FIP-U/mg) were obtained from Merck (Darmstadt, Germany). Deionized water (Milli-Q, 18.2 MΩ·cm) was used in all experiments in this study. All materials were used as received.

### 2.2. Preparation of Mesoporous Silica Nanoparticles (MSN)

MSN were synthesized using a previously reported method with slight modification [37,38]. Briefly, for typical synthesis, 6 mmol of TEOS was added to a solution containing CTAC (1.5 mmol), deionized water (150 mL), and NaOH (1.1 mL, 2 M) at 80 °C; the mixture was then stirred for 2 h. Silica nanoparticles were obtained by centrifugation at 11,000 rpm for 15 min and washed with ethanol and water. The surfactant was removed using an acid ethanolic solution (1.2 M HCl final concentration) at 80 °C for 18 h. This procedure was performed twice. Finally, the product was thoroughly washed with ethanol and water. 

### 2.3. Preparation of Amino-Functionalized Mesoporous Silica Nanoparticles (AMSN)

MSN were surface amino-functionalized using previously reported procedures [21]. Briefly, 300 mg of MSN were suspended in ethanol and 500 µL of APTES were added; then the mixture was stirred at 40 °C for 12 h. The AMSN were obtained by centrifugation at 11,000 rpm for 15 min and washed with ethanol.

### 2.4. Immobilized of Rosmarinic Acid onto Amino-Functionalized Mesoporous Silica Nanoparticles (Nano-RA)

RA was conjugated onto silica nanoparticles by coupling its –COOH group to the –NH_2_ groups of AMSN using the EDC/NHS coupling agents, according to previously reported procedure [21]. To activate the RA, 0.14 mmol of RA, 1.1 mmol of EDC and 0.56 mmol of NHS were suspended in water and homogenized for 20 min under N_2_ atmosphere. Then, the mixture was added dropwise to a suspension of AMSN, and the reaction was allowed to proceed with gentle stirring at room temperature under an N_2_ atmosphere for 6 h. The resulting product was collected by centrifugation and thoroughly washed with ethanol and water to remove all by-products and/or unreacted reagents. The RA conjugation was quantified using an indirect HPLC method. 

### 2.5. Morin Hydrate-Loading Procedure

For MH loading in the antioxidant nanosystem, an impregnation/solvent evaporation technique was used [39]. To this end, 25 mg of nano-RA (5 mg/mL suspension) were mixed with 25 mg of MH in a round bottom flask using methanol as solvent, and the suspension was stirred for 12 h. Then, the solvent was slowly evaporated at 25 °C using a rotary evaporator to 1 mL to produce a gradually increase concentration gradient of MH between the external solution and the nanoparticles, in order to favor the uptake of MH into mesopores. Subsequently, the MH loaded nano-RA samples were collected by centrifugation and the supernatant quantified by HPLC to determine the drug loading percentage (% DL), according to the following Equation (1):

(1) $$\%DL=\frac{MH_{initial}-MH_{free}}{Total\;weight}\times 100$$

where, *MH_initial_* and *MH_free_* are the amounts of morin initially added to the preparation of MH-loaded nano-RA and the amount of non-impregnated morin determined by the HPLC method, respectively. *Total weight* corresponds to the total amount of the antioxidant nanosystem and morin in the final preparation.

### 2.6. Characterization

The zeta potential and hydrodynamic diameter of the nanoparticles were obtained using a Malvern Zetasizer Nano ZS90 (Malvern, UK) with a detection angle of 173° and equilibration time of 120 s. Each measurement was performed three times. For the determination of zeta potentials, the nanoparticles were suspended in deionized water. Transmission electron microscopy (TEM) images were taken on a Hitachi HT7700 model microscope, with an accelerating voltage of 120.00 kV. N_2_ adsorption–desorption isotherms were measured on a Quantasorb system Model QS-17 (Quantachrome Instrument, Boynton Beach, FL, USA) and specific surface area was obtained using the multipoint BET method. Desorbed volume was obtained in the relative pressure range of 0.05–0.95. All samples were outgassed for 2 h at 110 °C under N_2_ flow. Fourier-transform infrared (FT-IR) spectra were recorded on a Nicolet iS5 instrument (Thermo Scientific, USA) with 4-cm^−1^ resolution, between 4000 and 500 cm^−1^, and the final spectrum corresponds to an average of 16 scans. The physical state of the pure MH, pure RA, MH-loaded nano-RA, and physical mixture of MH, RA, and nano-RA samples were conducted by differential scanning calorimetry (DSC). DSC curves were obtained on SETARAM 131 Evo apparatus (Setaram instrumentation K&P technologies, France). All samples were accurately weighted (1–4 mg), sealed in aluminum pans, and heated from 30 to 320 °C at a heating rate of 10 °C·min^−1^.

Determination of RA grafting onto nanoparticles was also quantified using an HPLC indirect method previously reported with slight modifications [40]. Chromatographic analysis was performed on an HPLC LC-20AT (Shimadzu, Tokyo, Japan), equipped with a model LC-20ATX2units pump, a Sil-20A autosampler, a CTO-20A column oven, an SPD-M20A diode array detector, and an InertSustain C18 column (4.6 × 250 mm, 5-µm particle size). Gradient elution was performed using a mobile phase consisting of a mixture of phase A (0.6% acetic acid solution) and phase B (acetonitrile). The starting mobile phase consisted of 80% A and 20% B for 4 min; then, from 5 to 15 min, the mobile phase was 40% A and 60% B, and finally from 16 to 17 min, the mobile phase was 80% A and 20% B; the flow rate was 1.0 mL/min. The sample injection volume was 20 µL, run time was 17 min, and retention time was 12.9 min. A PDA detector was set at 332 nm. Determination of MH-loaded nano-RA was also quantified using the same HPLC LC-20AT (Shimadzu, Japan) instrument described above, equipped with a Kromasil C8 column (150 × 4.6 mm). For MH analysis, mobile phase consisting of TFA 0.1%:acetonitrile (70:30, *v*/*v*) was pumped at a flow rate of 1 mL/min and sample injection volume was 20 µL. The run time was 16 min, retention time was 5.45 min, and the PDA detector was set at 355 nm. Results for all the samples were obtained by triplicate analysis.

### 2.7. Antioxidant Activity Evaluation (DPPH^●^)

DPPH^●^ free radical scavenging activity of nano-RA was evaluated according to previously reported methods [41]. Briefly, aliquots of RA, either free or nanoparticle-bound, with varying final concentrations (0.43, 1.1, 2.2, 4.3, 6.5 and 10.8 µg/mL) were added to a 5-mL volumetric flask and mixed with 1 mL of a DPPH^●^ solution freshly prepared in methanol (50 µM final concentration). Mixtures were stirred in the dark at room temperature for 20 min, then centrifuged, and the supernatant was measured spectrophotometrically at 515 nm using an Agilent UV/vis spectrophotometer (Agilent 8453 model, Santa Clara, CA, USA). The radical-scavenging activity (%RSA) was calculated according to the following Equation (2):

(2) $$\%RSA=\left(\frac{A_{0}-A_{1}}{A_{0}}\right)\times 100$$

where $A_{0}$ is the control absorbance (DPPH^●^ solution only) and $A_{1}$ is the absorbance of the sample after treatment. 

### 2.8. In Vitro Morin Hydrate Release Analysis

The in vitro release studies were carried out by dialysis technique, adapting previously reported procedures [42], using simulated gastric fluid (SGF) (pH 1.2 ± 0.1) and simulated intestinal fluid (SIF) (pH 6.8 ± 0.1) containing pepsin and pancreatin, respectively, as biorelevant media [43]. In each experiment, the dialysis bag was soaked and then filled with 4 mL of each sample containing an equivalent amount of 1 mg of MH. Subsequently, the dialysis bag was immersed in a tube containing 40 mL of biorelevant medium and kept at 37 °C under stirring at 100 rpm. Sink conditions were ensured throughout the experiments. At predetermined time intervals, aliquots of 1 mL were withdrawn from the release medium and immediately replaced with 1 mL of fresh medium. Each aliquot was filtered with a 0.22 µm syringe filter and MH content was determined by HPLC. All the measurements were performed in triplicate and the data expressed as cumulative percentage of MH released versus elapsed time (h). 

The MH release data from the nanoparticles were fitted using different kinetic models, including zero-order (Equation (3)), first-order (Equation (4)), Higuchi (Equation (5)), and Korsmeyer-Peppas (Equation (6)) models [44]:

(3) $$Q_{t}=Q_{0}+K_{0}t$$

(4) $$\ln Q_{t}=\ln Q_{0}+K_{1}t$$

(5) $$Q_{t}=K_{H}\sqrt{t}$$

(6) $$\frac{Q_{t}}{Q_{\infty }}=K_{KP}t^{n}$$

where *Q*_0_ and *Q_t_* is the initial amount of MH in the solution and the amount of MH dissolved at *t* time, respectively. *Q*_t_/*Q*_∞_ is the fractional release of drug (MH). *K*_0_ is the zero-order constant, *K*_1_ is the first-order constant, *K*_H_ is the Higuchi constant, *K*_KP_ is the Korsmeyer-Peppas constant, and *n* is the diffusional or release exponent.

### 2.9. Statistical Analysis

Data are presented as mean ± SD of *n* independent experiments. Data sets were analyzed by *R*^2^ parameter and Student’s *t*-test, followed by a Holm–Sidak post-hoc test, where applicable. A *p*-value < 0.05 was considered significant. The DDSolver® add-In (Microsoft Excel) program and GraphPad Prism software version 6.01 (San Diego, CA, USA) were used for statistical analysis. 

## 3. Results

**Figure 1:**
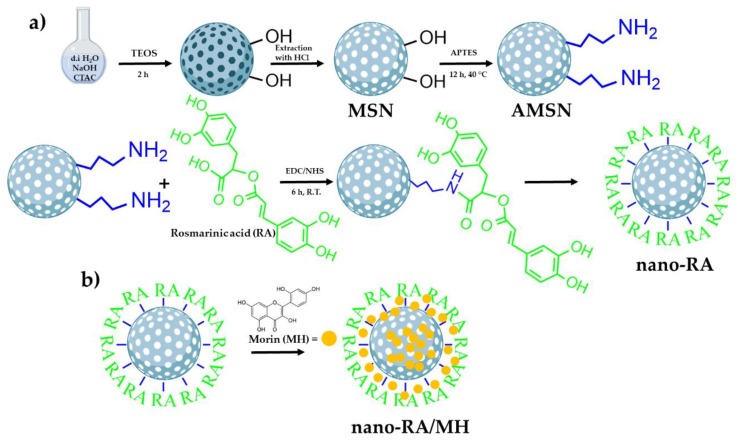
Schematic representation of the preparation of morin-loaded nanoantioxidants (nano-RA/MH). (**a**) Schematic path to obtain the antioxidant nanocarrier (nano-RA) and (**b**) schematic representation of the loading of morin in the nano-RA (nano-RA/MH).

In this work, an antioxidant material consisting of a mesoporous silica nanostructure and rosmarinic acid (nano-RA) was developed and characterized, to unravel the release mechanism of bioactive compounds from this drug delivery model system. Recently, we reported the preparation of an antioxidant-nanosystem based on caffeic acid attached to silica nanoparticles, which showed the ability to protect oxidant-sensitive molecules [21]. Employing similar protocols to those previously reported, the RA, an ester derived from a hydroxycinnamic acid, was successfully attached onto amino-functionalized mesoporous silica nanoparticles through an amide bond between the carboxylic acid of RA and APTES amino group on the surface of the nanoparticles (Figure 1a). 

**Figure 2:**
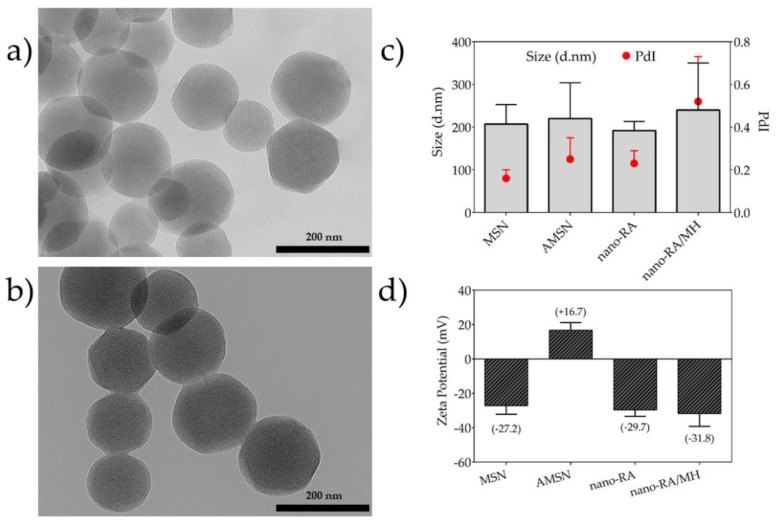
Transmission electron microscopy (TEM) micrographs and particle size distribution. (**a**) TEM image of bare mesoporous silica nanoparticles (MSN), (**b**) TEM image of rosmarinic acid–functionalized silica nanocarrier (nano-RA), (**c**) hydrodynamic diameter and polydispersity index (PdI) of nanomaterials by dynamic light scattering (DLS) measurement, and (**d**) zeta potential of nanomaterials.

According to previous reports regarding the reaction time to graft a polyphenol by EDC/NHS coupling in polymeric matrices, 6 h of reaction time is sufficient to successfully attach RA on the surface [16,21,45,46]. In concordance, the grafted amount was 216.2 mg of RA per gram of nanoparticle, yielding an 88% (Table 1). After the grafting process and using bare nanoparticles (MSN) as control, MH was loaded into nanoparticles using the impregnation/solvent evaporation technique (Figure 1b). The MH-loaded was 9.4% *w*/*w* and 23.1% *w*/*w* for MSN/MH and nano-RA/MH, respectively (Table 1); these values are expected due to MH being incorporated into the MSN pores and a small amount adsorbed on the surface. In the nano-RA, MH was also incorporated into the pore and, in addition, interacted with moieties of RA, producing a greater amount of MH adsorbed compared to bare MSN. The hydrodynamic diameter of all nanomaterials was measured (Figure 2c). In general, the size does not vary significantly with the different functionalization steps, maintaining an average of 205 nm with a good polydispersity index lower than 0.3 (Figure 2c, right *y* axis), except in the case of nano-RA/MH, where the average hydrodynamic diameter increases up to 250 nm with a PdI value of 0.52, probably due to the possible adsorption of morin in multilayers. This behavior has been previously reported by our group and is related to the coating properties of morin [47,48]. Nevertheless, any change in size was negligible and the zeta potential values (Figure 2d) suggest a suitable colloidal stability. In addition, the zeta potential values reveal change in surface charge from bare mesoporous silica nanoparticles (−27 mV) to particles functionalized with amino groups (+17 mV), then to particles with RA immobilized on surface through an amide bond (−30 mV), and finally, to silica nanospheres with MH incorporated both in the pores and onto the surface (−32 mV). 

The homogeneity of nanosystems was corroborated through the morphological features of nanomaterials obtained by TEM images (Figure 2a,b), where the particles were monodisperse porous spheres with a size of ca. 150.9 ± 18.6 nm; the surface functionalization does not affect the morphological features of studied nanosystems. In addition, the TEM images revealed that the nanoparticles are characterized by regular and well-ordered parallel pore channels, with a pore diameter ~3.1 nm, in agreement with those reported by other authors [37]. For bare MSN and nano-RA, the BET surface area determined were 840 m^2^·g^−1^ and 267 m^2^·g^−1^ (Table 1); this change, as expected, suggests that the RA molecules are linked in both the pore and outer surface of the nanoparticles. It is expected that this disposition of the RA molecules can affect the amount of MH loaded and its subsequent release, due to a greater interaction between RA/MH than MH/MSN. In fact, these results are consistent with the high drug loading of MH (23% *w*/*w*) in nano-RA compared to bare MSN (9% *w*/*w*). 

**Figure 3:**
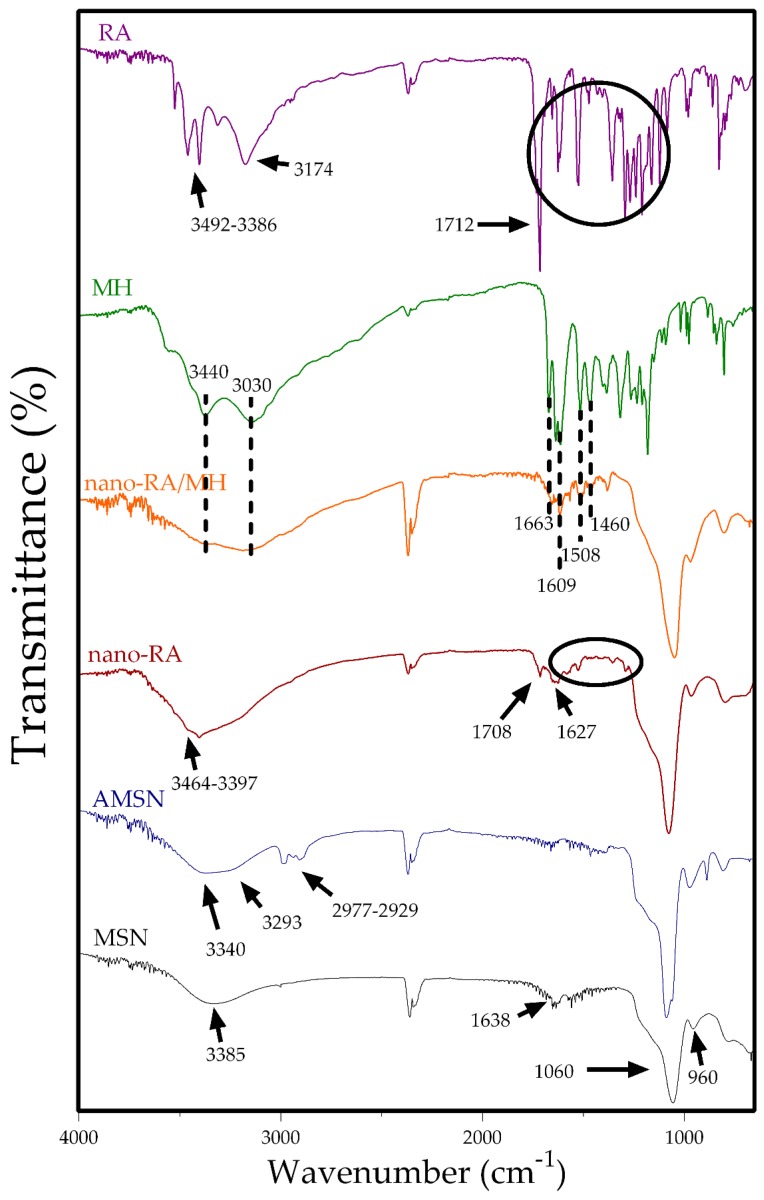
Fourier-transform infrared (FT-IR) spectra of MSN (black line), AMNS (blue line), nano-RA (red line), nano-RA/MH (orange line), MH (green line), and RA (purple line).

In order to evaluate the surface functionalization of nanoparticles, the FTIR spectra of all nanosystems and the standard polyphenols were recorded (Figure 3). MSN spectrum (black line) exhibit a broad band at 3385 cm^−1^ corresponding to adsorbed water and hydroxyl stretching of the silanol group, consistent with the presence of two characteristic peaks at 1638 cm^−1^ (H–OH twisting band) and 960 cm^−1^ attributed to Si–O band stretch of surfaces Si–OH groups. Furthermore, a broad characteristic band at 1060 cm^−1^ corresponding to stretching vibration of siloxane groups is also present.

After amino-functionalization, AMSN (blue line) showed four important peaks at 3340, 3293, 2977, and 2929 cm^−1^, where the former two are related to NH_2_ stretching vibration and the last two peaks are attributed to the C–H stretch of aminopropyl chain of APTES [49]. The RA spectrum (purple line) indicates the main peaks at 3492 and 3386 cm^−1^ attributed to stretching of free –OH groups of RA, and the peaks at 3174 and 1712 cm^−1^ are due to –OH stretching and –C=O stretching vibration of carboxylic acid, respectively. Also, the spectrum showed the characteristic fingerprints of RA, where the principal peaks at 1648 cm^−1^ and 1517 and 1471 cm^−1^ were assigned to –C=O stretching of conjugated carbonyl group and stretching related to the aromatic ring, respectively. The nano-RA spectrum (red line) also exhibits several of the characteristics peaks in the fingerprint zone (1521 and 1458 cm^−1^) and the presence of a broad band (3510–3100 cm^−1^) due to –OH stretching and two weak peaks at 3464 and 3397 cm^−1^ due to free –OH of RA. Additionally, two differences are crucial when comparing nano-RA spectrum to the AMSN and RA spectra. First, the absence of the peak corresponding to vibration of the –OH of carboxylic acid, and secondly, the presence of two new bands at 1708 and 1627 cm^−1^ attributed to (–C=O) amide I and (–C-N) amide II, due to the formation of amide bond between NH_2_ of APTES and carboxylic acid of RA, confirming the successful grafting of RA onto the surface of mesoporous silica nanoparticles [16]. On the other hand, in the MH spectrum (green line) were highlighted bands at 3440–3030 cm^−1^ of the –OH group of morin, at 1663 cm^−1^ of –C=O of the carbonyl group in the C ring of morin, and the peaks at 1609, 1508 and 1460 cm^−1^ attributed to C=C stretching vibration in the aromatic ring. The same previous peaks are present in the nano-RA/MH spectrum (orange line), suggesting the presence of morin in the surface of nanomaterial (considering the external zone of pores).

**Figure 4:**
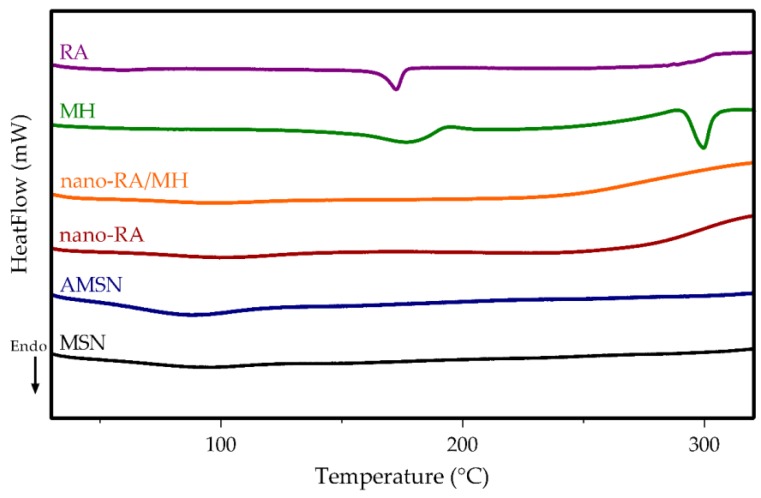
DSC thermogram of MSN (black line), AMSN (blue line), nano-RA (red line), nano-RA/MH (orange line), MH (green line), and RA (purple line).

Differential scanning calorimetry (DSC) analysis was using to investigate the physical state of MH after being incorporated in nanosystems (Figure 4), and the DSC thermograms were designated as pure MH (green line), pure RA (purple line), MSN (black line), AMSN (blue line), nano-RA (red line), and nano-RA/MH (orange line). 

The curve corresponding to pure MH displays a sharp endothermic peak at 296 °C, which corresponds to the intrinsic melting point [50]. However, this peak did not appear in the nano-RA/MH sample, indicating that the MH was in a noncrystalline state when incorporated into the nanosystem, both in the pore and onto the surface interacting with the grafted RA; this molecular interaction favors the solubility of MH. Additionally, pure RA displays a sharp endothermic peak at 171 °C [51], but this peak, as expected, cannot be observed in the curve corresponding to nano-RA or the nano-RA/MH, due to the covalent interaction between RA and the surface of the mesoporous silica nanoparticles.

**Figure 5:**
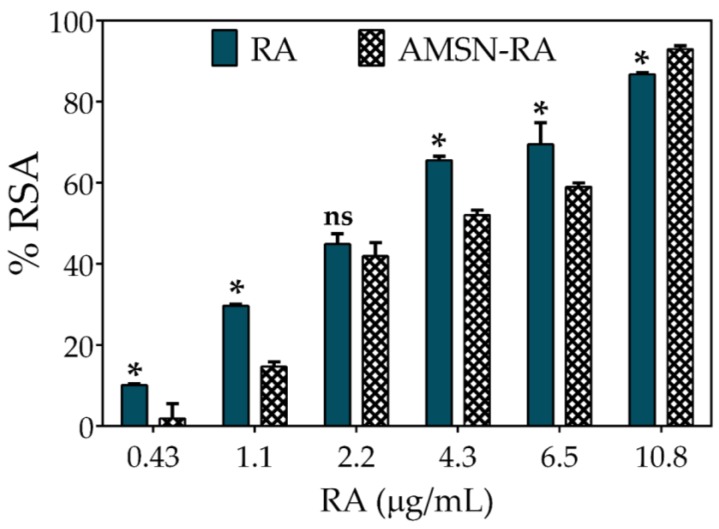
Antioxidant activity comparison between RA (rosmarinic acid standard) and nano-RA against DPPH^●^ expressed as percentage of radical scavenging activity (%RSA) versus RA concentration. Results are reported as means ± SD (*n* = 3). *p < 0.05; ns, not significant.

The DPPH^●^ assay is an easy-to-use, low cost, and fast method to screen antioxidant activity (number of reduced DPPH^●^ per antioxidant mole); it has been widely used by several authors to evaluate this activity of polyphenols incorporated in silica or polymeric matrices [16,52,53,54]. In the present work, we evaluated the antiradical activity expressed as radical scavenging activity percentage (%RSA) versus RA concentration of free RA and nano-RA, in the concentration range of 0.43–10.8 µg/mL (Figure 5). As the concentration of free RA increases, the %RSA increases, reaching a 50% of DPPH^●^ deactivation approximately at a concentration of 2.5–3 µg/mL, which is consistent with literature reports [55,56,57]. 

Xie and Schaich studied the kinetics patterns of different phenol reactions with DPPH^●^, and classified them into five groups. According to the reactivity, they were denominated as instantaneous (group 1), very fast (group 2), moderate (group 3), slow (group 4), and no reaction (group 5). Considering this work, RA reduces DPPH^●^ through a moderate kinetic pattern, where it first acts by a single electron transfer (SET) mechanism and as the reaction progresses, the hydrogen atom transfers (HAT) and steric hindrance or mixed mechanisms appear [58,59]. On the other hand, nano-RA also shows a similar concentration-dependence activity but with a slight decrease in the %RSA, exhibiting a 50% of DPPH^●^ deactivation at RA nominal concentration of ~4.3 µg mL^−1^. This behavior could be explained by the immobilization of RA on the surface of nanoparticles and the tendency of DPPH^●^ to adsorb onto nanoparticles; when combined, they are a limitation to the free diffusion of involved molecules, hindering antioxidant activity. This hampering effect of nanoparticles has a pivotal role, since the radical site of DPPH^●^ is protected inside a reaction cage formed by nitro groups oriented above and below it, by an ortho H atom on each of the phenyl rings, and by twisting of the ring [59]; therefore, considering this orientation, the steric accessibility is crucial for DPPH^●^ deactivation. Thus, in systems where nanoparticles limit accessibility, an attenuation in the activity can be expected. However, although the antioxidant activity of RA decreases slightly, the nanomaterial shows a high antioxidant activity; this behavior has been reported by other authors [16,21,52]. These results confirm that the RA scaffold is able to scavenge a free radical when favorably oriented on the surface of nanoparticles.

**Figure 6:**
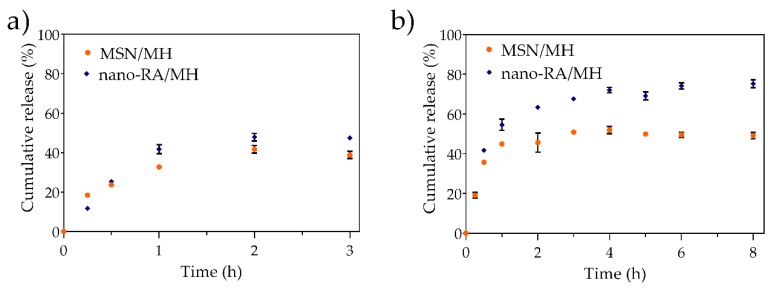
In vitro release profile of morin loaded in MSN (MSN/MH) and nano-RA (nano-RA/MH), in (**a**) simulated gastric fluid and (**b**) simulated intestinal fluid, at 37 °C.

As mentioned earlier, MH is a flavonoid with several reported pharmacological activities; however, its poor aqueous solubility produce low oral bioavailability [29], limiting its pharmaceutical applications. As many other drugs, polyphenol absorption occurs mainly in the small intestine [60]; however, in the case of MH, the information about its oral delivery is limited. Nevertheless, pharmacokinetic studies in animal models by different authors have reported that MH absorption occurs mainly in the small intestine and the colon [30,31,61]. Some of these studies suggest that to improve the oral bioavailability of MH, a BCS class IV drug, it is crucial to avoid both efflux and intestinal metabolism. Thus, the release of more or less MH in the absorption site would not be a preponderant factor, since the process still depends on the physicochemical properties of the molecule in the biological milieu. However, it is important to consider that although the release mechanism of some large and small molecules from silica matrices has been widely reported [62], to our knowledge, there are no reports on the release mechanism of polyphenols from systems called nanoantioxidants [13,63] based on silica. Unveiling this mechanism could anticipate biological behavior and optimize the design of pharmaceutical formulations to improve oral bioavailability. In addition, the polyphenolic surface of these novel nanosystems, also classified as bioinspired carriers, has been reported as a component that could enhance the biocompatibility, biodegradability, and targeting properties of the silica materials [64]. Thus, the implications of the polyphenol-functionalized nanoparticle on the drug release need to be evaluated. Therefore, the in vitro release profile of MH-loaded antioxidant-nanosystem (nanoantioxidant) in two biorelevant media were studied (Figure 6). 

In the first stage (Figure 6a), the nanosystem was evaluated in simulated gastric fluid (SGF, pH 1.2) where the release of MH was <40% both in bare nanoparticles and antioxidant-nanosystem, reaching a plateau at 2 h. In the second stage (Figure 6b), the nanosystem was evaluated in simulated intestinal fluid (SIF, pH 6.8), where the release of MH from bare nanoparticles reached the maximum cumulative release at 2 h (<50%). On the other hand, the release of MH from the antioxidant nanosystem was greater than that from bare nanoparticles, reaching a maximum MH release of 74% at 4 h; the system then showed a sustained release until the end of the experiment. Other authors have reported the difficulty of administering high doses of morin orally, because it tends to form a saturated solution in the intestinal tract, hindering the drug dissolution process. In this context, the sustained release of a drug from the antioxidant nanosystem could be beneficial. When the nanoparticles are in SGF, no significant difference in the low sustained release of MH from MSN/MH and nano-RA/MH systems are observed, and the release of MH from the nano-RA/MH is slightly higher. This could be explained because at acidic pH of SGF, both the MH and RA species are protonated, which does not favor the solubility of MH; therefore, only a small amount of MH diffuses from the pore to the medium, but a slightly higher amount of MH is released from the antioxidant-nanosystem, due to the adsorbed MH on the surface that interacts with RA. A different situation is observed when the nanoparticles are in SIF. In this medium, the RA and MH are partially ionized, which favors both the solubility of the MH and its repulsion with the polyphenolic surface, triggering a greater release; in particular, the antioxidant-nanosystem produces a higher release than the bare nanoparticles due to the MH being adsorbed on the surface, enhanced by the RA-MH interaction, and the MH-loaded in the pores.

According to the literature, the drug release process comprises of four pivotal stages: (i) entry of the release medium into the material matrix, in response to the osmotic pressure produced by the concentration gradient of the drug between the solution and the particle surface; (ii) drug dissolution in the release medium; (iii) drug diffusion through the particle matrix due to a concentration gradient; and (iv) drug diffusion and transport within the release medium [65,66]. Hence, to understand the specific MH release mechanism from the nanoparticles, the data were fitted according to four kinetic models commonly used and suitable to analyze the release of drug from silica nanoparticles. In order to discriminate which mathematical model best fits the kinetic data, the R^2^ parameter was used. The release data were best fit by Higuchi and Korsmeyer-Peppas models, independent of the biorelevant media tested (Table 2), demonstrating that MH release from nanosystems involves a diffusion process. A deeper insight into the release mechanism was performed using the release exponent value (*n*) according to the Korsmeyer-Peppas model, which is classified in different categories [67]. For spherical materials, if *n* < 0.43, the drug release mechanism follows a pseudo-Fickian diffusion (commonly for polydisperse systems); *n* ~ 0.43 indicates Fickian diffusion; when 0.43 < *n* < 0.85, anomalous (non-Fickian) transport is involved; if *n* ~ 0.85 indicated non-Fickian Case-II transport; and finally, if *n* > 0.85 the predominant drug release mechanism is a Super Case II transport. 

In particular, the *n* values for MH release from bare nanoparticles in SGF and SIF were 0.42 and 0.43, respectively, which indicates a Fickian diffusion mechanism. These results are in concordance with other reports for porous matrix structures [62,66]. Otherwise, it is interesting to note that when the antioxidant (RA) is on the nanoparticles surface, the *n* values in SGF and SIF change to 0.68 and 0.54, respectively, indicating an anomalous non-Fickian transport. This could be explained by considering the interaction between RA and MH on the surface and the layer-like arrangement on the nanoparticles. Tran and Lee reported similar results regarding the change in the diffusion mechanism from bare nanoparticles to surface functionalized layer-like arrangement nanoparticles [68]. 

It is well known that in the Fickian diffusion, the solvent diffuses into the matrix at a high rate and polymeric relaxation has a low rate, inducing the formation of a gradient of solvent penetration. On the other hand, in the anomalous transport, the rate of solvent diffusion and the polymeric relaxation possess similar magnitudes [69]. Therefore, this anomalous transport is characterized by a coupling of the diffusion and the matrix erosion mechanism, i.e., the release is controlled by more than one process. In this sense, several authors have reported the degradation of silica matrix in different biorelevant media and at different average times; for example, 3 days [70], 8–12 days [6], 8 h [71], and 24–96 h [72]. On the other hand, Braun et al. reported no degradation of silica matrix when exposed to SGF for 6 h [73]. In any case, the degradation process as a basis for explaining the change in the diffusion mechanism is not considered in our work, because of the short experimental times involved. Thus, to explain the behavior of MH release when the polyphenol RA is grafted in the nanoparticles, it is necessary to consider a possible layer-like arrangement on the surface. Mainly, the –OH and -C=O groups belonging to MH and RA can interact through hydrogen-bonding and due to the coating capacity of MH, form more than one surface layer that is not removed with washing after the synthesis [47,74,75]. As mentioned earlier, the release occurs in the SGF and SIF media, but when the pH increases, the MH and RA molecules are partially deprotonated and the electrostatic repulsive force between them facilitates the release, and the outer polyphenolic layers are removed, to a greater extent than at acidic pH, due to the process of MH desorption. In addition, it is noteworthy that no significant burst release was observed in the release profile. Thereby, the release mechanism should take place by two processes: (i) the diffusion through the pores and (ii) the ionization of MH and subsequent slow desorption. The last process is crucial because it resembles the erosion mechanism mentioned above, hindering the transport and disrupting the normal Fickian diffusion from pores, explaining the change in the mechanism from Fickian diffusion to anomalous non-Fickian transport both in SGF and SIF media, for the nano-RA system.

Considering that anomalous transport involves both the Fickian diffusion and relaxation/erosion mechanisms, it is interesting to identify the main process in the MH release from the antioxidant-nanosystem. To achieve this, the empirical model proposed by Nikolaos Peppas and Jennifer Sahlin was used [67]. This model aims to calculate the approximate contributions of the diffusional and relaxation mechanisms in an anomalous solute release process, considering them additives according to the following equation:

(7) $$\frac{M_{t}}{M_{\infty }}=k_{1}t^{m}+k_{2}t^{2m}$$

where, the first term of the right-hand side is the Fickian contribution and the second term is associated with the relaxation release. The coefficient *m* is the purely Fickian diffusion exponent. By rewriting and rearranging Equation (7), it is possible to calculate the percentage of MH release due to the Fickian mechanism, *F*, expressed as follows:

(8) $$F=\frac{1}{1+\frac{k_{2}}{k_{1}}t^{m}}$$

Considering this expression, the ratio between relaxation (*R*) and Fickian (*F*) contributions is expressed as:

(9) $$\frac{R}{F}=\frac{k_{2}}{k_{1}}t^{m}$$

If: (i)$\;\frac{R}{F}<1$, the Fickian diffusion dominates the process, (ii)$\;\frac{R}{F}=1$, the release involves both diffusion and relaxation/erosion process, and (iii)$\;\frac{R}{F}>1$, relaxation/erosion dominates the release process [76]. Our results show that the data fit well to the Peppas-Sahlin model (*R*^2^_SGF_ = 0.90 and *R*^2^_SIF_ = 0.91), where $\frac{k_{2}}{k_{1}}$ was 0.18 and 0.074 for MH release in the SGF and SIF media, respectively; in addition, the $\frac{R}{F}$ ratio was < 1 over the entire time range in both the biorelevant media studied. These results clearly show that despite the MH release being a coupling of Fickian diffusion and relaxation/erosion, the former is the dominant mechanism. In fact, the contribution of Fickian diffusion for MH release in SGF was more than 80% and the approximate contribution in the SIF was higher than 90%.

## 4. Conclusions

A hybrid nanoantioxidant carrier based on porous silica nanoplatform with rosmarinic acid antioxidant immobilized on its surface was successfully development and characterized. Morin flavonoid was incorporated on the antioxidant nanocarrier by the impregnation/solvent evaporation technique with high drug loading (23% *w*/*w*) compared to bare MSN (9% *w*/*w*). The polyphenol–coated nanosystem maintains high antiradical activity of rosmarinic acid, showing the potential antioxidant activity of this nanosystem to protect different molecules from oxidation. Morin release profile was assessed in two biorelevant media, where the results show that the antioxidant nanosystem is capable of releasing more amount of morin at pH 6.8 than at acidic pH. This suggests that drug release will be favored at a more alkaline pH, such as intestinal or colon segment, due to the repulsive force between the drug and the polyphenol layer on the surface. Kinetic studies show that the polyphenolic layer promotes a disturbance in the release mechanism compared to bare mesoporous silica nanoparticles, changing the Fickian diffusion to an anomalous non–Fickian transport. Nonetheless, the Fickian diffusion contribution is more than 80% of the release mechanism, suggesting that the polyphenolic surface could be tailored to favor of one or another release mechanism. Together, these results indicate the presence of an antioxidant nanocarrier for potential drug delivery application by incorporating it into a pharmaceutical formulation, which enhances the solubility of a drug and controls its release at the intestinal level. 

## Figures and Tables

**Table 1:** Summary of physical data of the tested nanomaterials.

Nanosystem	*S*_BET_ (m^2^/g) ^a^	Grafting (mg RA/g NP) ^b^	%MH Loading (*w*/*w*) ^b^	Theoretical %MH Loading (*w*/*w*)
MSN	840 ± 10	-	-	-
nano-RA	267 ± 1.0	216.2 ± 3.8	-	-
MSN/MH	-	-	9.4 ± 0.8	50
nano-RA/MH	-	-	23.1 ± 3.5	50

^a^ Determined using the multipoint BET method. ^b^ Determined by HPLC methodology. BET method–Brunauer-Emmett-Teller method; SBET–BET surface area; HPLC–High-performance liquid chromatography; MSN–Mesoporous silica nanoparticles; MSN/MH–morin-loaded mesoporous silica nanoparticles; nano-RA–rosmarinic acid-functionalized silica nanocarrier; nano-RA/MH – morin-loaded rosmarinic acid-functionalized silica nanocarrier.

**Table 2:** Kinetics parameters of the release profile of nanosystems in simulated gastric fluid and simulated intestinal fluid.

Medium Release	Nanosystem	Kinetic Model							
		Zero-order		First-order		Higuchi		Korsmeyer-Peppas	
		*K*_0_ (mg h^−1^)	*R* ^2^	*K*_1_ (h^−1^)	*R* ^2^	*K*_H_ (mg *t*^−0.5^)	*R* ^2^	*n*	*R* ^2^
SGF	MSN/MH	0.13	0.96	0.44	0.91	0.26	0.99	0.42	0.99
	nano-RA/MH	0.19	0.83	0.69	0.73	0.39	0.92	0.68	0.91
SIF	MSN/MH	0.03	0.422	0.08	0.37	0.33	0.88	0.43	0.88
	nano-RA/MH	0.05	0.56	0.11	0.45	0.36	0.86	0.54	0.88
